# Supplementary material for: COVID-19 Continues to Burden General Practitioners: Impact on Workload, Provision of Care, and Intention to Leave
Source: Healthcare (Basel). 2023 Jan 20;11(3):320. doi: 10.3390/healthcare11030320 (PMC9914234; doi:10.3390/healthcare11030320)
Supplement: Supplementary file 1 [file healthcare-11-00320-s001.zip › healthcare-2124887-supplementary.pdf]

Supplements

Table S1. GP questionnaire translated into English (baseline 2021).

**1. Demographics**					
Age:	__ __ years	Sex:	□ female	□ male	□ diverse
Medical specialist for: *multiple responses possible*	□ General Practice<br>□ Internal Medicine		□ Others ________________		
Number of cases per quarter:	□ ≤ 700	□ 701-1000	□ 1001-1500	□ ≥ 1501	□ No answer
Number of health insurance approved physicians:				__ __	
Practice structure:	□ Single pratice	□ Practice sharing	□ Joint practice	□ Medical center	
Catchment area of the practice: *multiple responses possible*	□ City	□ Small town	□ Rural		
Is your practice a COVID focal point practice?		□ Yes	□ No		

**2. Patients with acute COVID-19 and long COVID treated in the practice**	
How often do you approximately treat patients with acute COVID-19 in your practice?<br>□ Daily<br>□ Weekly<br>□ Monthly<br>□ Less than monthly<br>□ Never	
How often do you approximately treat patients with long COVID (symptoms lasting between 4-12 weeks) in your practice?<br>□ Daily<br>□ Weekly<br>□ Monthly<br>□ Less than monthly<br>□ Never	
How often do you approximately treat patients with long COVID (symptoms lasting more than 12 weeks) in your practice?<br>□ Daily<br>□ Weekly<br>□ Monthly<br>□ Less than monthly<br>□ Never	
How many patients with long COVID (symptoms lasting between 4-12 weeks) are you currently treating in your practice?	_ _
How many patients with long COVID (symptoms lasting more than 12 weeks) are you currently treating in your practice?	_ _
How able do you feel to address the medical and/or psychological needs of patients with acute COVID-19 or long COVID during consultations on a scale from 1 = "not able" to 10 = "fully able"?	

□<br>1	□<br>2	□<br>3	□<br>4	□<br>5	□<br>6	□<br>7	□<br>8	□<br>9	□<br>10

**3. Problems and needs with respect to patients with long COVID**
What problems do you currently encounter related the treatment of patients with long COVID? (*multiple responses possible*)<br>□ Diagnosis is difficult<br>□ Unspecific symptoms<br>□ Uncertainty regarding medications<br>□ Long course of disease<br>□ No guidelines available<br>□ Other: ________________________

Which support services do you consider particularly relevant regarding COVID-19? (*multiple responses possible*)<br>□ Specialized information on typical symptoms and their length<br>□ Exchange with colleagues on case reports and workshops<br>□ Online training<br>□ Diagnostic tools<br>□ Special therapies for patients with long COVID<br>□ Guidelines on long COVID<br>□ Referral options to specialized outpatient clinics<br>□ Other: _______ ________________

**4. Practice situation due to the pandemic**									
Compared to pre-pandemic times, my practice is visited by patients…					□ More frequently<br>□ As frequently<br>□ Less frequently				
Compared to pre-pandemic times, I need for patient consultations…					□ More time<br>□ As much time<br>□ Less time				
Compared to pre-pandemic times, my workload is…					□ Higher<br>□ Just the same<br>□ Lower				
Compared to pre-pandemic times, the economic situation of my practice…					□ Improved<br>□ Did not change<br>□ Worsened				
How much does the COVID-19 pandemic limit the provision of satisfactory medical care to other patients with chronic diseases (e.g., early diagnosis, disease management programs, preventive care) in your practice on a scale from 1 = “fully limited” to 10 = “not limited”?									
□<br>1	□<br>2	□<br>3	□<br>4	□<br>5	□<br>6	□<br>7	□<br>8	□<br>9	□<br>10

**5. General comment**
Is there anything else you would like to tell us about long COVID in your practice?

Table S2. GP follow-up questionnaire translated into English (follow-up 2022).

**1. Patients with acute COVID-19 and long COVID treated in the practice**									
How many patients with acute COVID-19 are you currently treating in your practice?									_ _
How many patients with long COVID (symptoms lasting between 4-12 weeks) are you currently treating in your practice?									_ _
How many patients with long COVID (symptoms lasting more than 12 weeks) are you currently treating in your practice?									_ _
How able do you feel to address the medical and/or psychological needs of patients with acute COVID-19 or long COVID during consultations on a scale from 1 = "not able" to 10 = "fully able"?									
□ 1	□ 2	□ 3	□ 4	□ 5	□ 6	□ 7	□ 8	□ 9	□ 10

**2. Current consultation reasons**	
**How many out of 100 patients are currently visiting for the following reasons:**	
Acute COVID-19 infection	_ _
Long COVID (4-12 weeks after diagnosis)	_ _
Long COVID (>12 weeks after diagnosis)	_ _
Other infections	_ _
Other acute reasons	_ _
Care of chronic diseases	_ _
SARS-CoV-2 vaccinations	_ _
Other vaccinations	_ _
Other reasons	_ _
For which services can you currently offer fewer appointments? (*multiple responses possible*)	□ Acute consultations<br>□ Care of chronically ill patients<br>□ Preventive cancer screenings<br>□ Preventive health check-ups<br>□ Self-payer services<br>□ Home visits<br>□ Nursing home visits
How many SARS-CoV-2 vaccine doses do you currently administer per week?	_ _

**3. Practice situation due to the pandemic**									
Compared to pre-pandemic times, my practice is visited by patients…									□ More frequently<br>□ As frequently<br>□ Less frequently
Compared to pre-pandemic times, I need for patient consultations…									□ More time<br>□ As much time<br>□ Less time
Compared to pre-pandemic times, my workload is…									□ Higher<br>□ Just the same<br>□ Lower
Compared to pre-pandemic times, the economic situation of my practice…									□ Improved<br>□ Did not change<br>□ Worsened
How much does the COVID-19 pandemic limit the provision of satisfactory medical care to other patients with chronic diseases (e.g., early diagnosis, disease management programs, preventive care) in your practice on a scale from 1 = "fully limited" to 10 = "not limited"?									
□ 1	□ 2	□ 3	□ 4	□ 5	□ 6	□ 7	□ 8	□ 9	□ 10
Have you considered quitting your job in the last 12 months?									□ No<br>□ Yes

	□ If yes, why? _______________

**4. General comment**
What do you currently wish for in your everyday working life? What support could reduce your workload?

Table S3. Characteristics of participating GPs at baseline and follow up (GPs in the follow-up survey also participated in the baseline survey).

	**Baseline 2021**	**Follow-up 2022**
n	143	51
Age	50.2±9.4	49.6±9.4
Sex	61.1%♀	56.9%♀
Medical specialist for*		
General Medicine	65%	64.7%
Internal Medicine	33.6%	33.4%
Others	4.9%	5.9%
Practice structure		
Single practice	61.5%	56.9%
Practice sharing	10.5%	13.7%
Joint practice	16.1%	15.7%
Medical center	11.9%	13.7%
Number of health insurance approved physicians per practice	1.5	1.5
Number of cases per quarter		
≤700	7.7%	7.8%
701-1000	24.5%	23.5%
1001-1500	39.2%	37.3%
≥1501	26.6%	31.4%
No answer	2.1%	0%
Catchment area of the practice*		
City	39.2%	41.2%
Small town	44.8%	45.1%
Rural	41.3%	43.1%

Note. Data are presented as mean, standard deviations, and percentage (n/n_{valid}). *, multiple responses possible.

Table S4. Other problems and needs related to the treatment of patients with COVID-19 or long COVID: content analysis of free text answers at baseline 2021.

**Major Category**	**Subcategory**	**n***	**%****
Problems related to treatment			
Lack of medical options		12	8.4
	Lack of medical specialists	7	4.9
	Lack of special outpatient clinics	4	2.8
	Lack of (specialized) rehab facilities	2	1.4
	Lack of therapies	1	0.7
Patient-related problems		10	7.0
	Somatization	3	2.1
	Secondary morbid gain	2	1.4
	Problems to draw a distinct line to (pre-existing) mental disorders	2	1.4
	Rehab not wanted	1	0.7
	Unknown prognosis	1	0.7
	Impatience by patients	1	0.7
	Loss of trust by patients	1	0.7
Structural problems		4	2.8
	Uncertainty caused by media	2	1.4
	Employers show no understanding	1	0.7
	Lack of time	1	0.7
	Legal risks by off-label use	1	0.7
Needs related to treatment			
Need for therapies		9	6.3
	Need for improved psychotherapy access	3	2.1
	Need for improved rehab access	3	2.1
	Support through complementary medicine	1	0.7
	Options for specialist referral	1	0.7
	Access to more medication	1	0.7
Need for information		6	4.2
	Workshops and online seminars by medical associations	2	1.4
	Improvements of current information	1	0.7
	More reports in scientific literature or podcasts	1	0.7
	Quality circles	1	0.7
	Central (scientific) contact center to objectify the findings	1	0.7
Individual needs		3	2.1
	More time	1	0.7
	Self-protection	1	0.7
	Self-reflection	1	0.7

Note. n***** = statements in this category, ** % = percentage of GPs who stated an insight from this category.

Table S5. GPs' reasons for wanting to leave their job (n = 13): content analysis of free text answers.

**Major Category**	**Subcategory**	**n***	**%****
Changes in work		8	61.5
	Increased workload	5	38.5
	More administrative than medical tasks	4	30.8
	Loss of control over practice routines	2	15.4
	Limited therapeutic freedom	1	7.7
	Frustrating educational work	1	7.7
	Hospital admissions for non-COVID patients difficult	1	7.7
	GP colleagues increasingly vaccinate and reduce routine care	1	7.7
Patients' attitudes		8	61.5
	Demanding patients	4	30.8
	Increasing irritability between patients and practice members	2	15.4
	Lack of solidarity	1	7.7
	Lack of communication	1	7.7
	Insecure patients	1	7.7
Public and political reasons		7	53.8
	Handling of the pandemic by politicians and health authorities	2	15.4
	Lack of support by politicians and health authorities	2	15.4
	Insufficient budgeting	1	7.7
	Constantly changing instructions	1	7.7
	Call to action but no delivery of vaccine doses	1	7.7
	Public discussion about effectiveness of different vaccines and their unequal distribution	1	7.7
	Public discussion about compulsory vaccination	1	7.7
	Public misinformation	1	7.7
	Closing of vaccination centers	1	7.7
Personal reasons		4	30.8
	Lack of leisure time	2	15.4
	Lack of family time	1	7.7
	Lower quality of life	1	7.7
	No joy in occupation	1	7.7
Changes in practice team		3	23.1
	Staff shortage	2	15.4
	Staff not vaccinated	1	7.7
	Ill-informed staff	1	7.7
	Lack of communication	1	7.7
	Staff demanding higher salary	1	7.7
Telemedicine		3	23.1
	Malfunctioning telecommunications systems	2	15.4
	Burden of increased telemedicine	1	7.7

Note. n* = statements in this category, ** % = percentage of GPs who stated an insight from this category.

Table S6. GPs' general comments on long COVID at baseline 2021 (n = 33): content analysis of free text answers.

**Major Category**	**Subcategory**	**n***	**%****
Long COVID in practice		28	84.9
	Urgent need for therapies and rehabilitation	10	30.3
	Strong psychological comorbidity	6	18.2
	Urgent need for specialists/ambulances	6	18.2
	Observation of severe cases in practice	4	12.1
	Observation of only mild cases in practice	4	12.1
	Need for increased access to psycho(somatic)therapies	4	12.1
	Long COVID comparable to other protracted infections	3	9.1
	Long COVID is affecting the whole family	2	6.1
	GPs are still in the learning curve	2	6.1
	Patients get upset about lack of therapies/knowledge	1	3.0
	Need for higher budget	1	3.0
	Predominantly young patients	1	3.0
	Reoccurrence of previous diseases through COVID-19	1	3.0
	Lack of criteria for recovery	1	3.0
	Symptoms subside undulatory	1	3.0
Research and politics		3	9.1
	Few scientific recommendations for action	2	6.1
	Few recommendations for action from the official side	1	3.0
	No support from health insurances	1	3.0
	Not enough research on long COVID	1	3.0
Media coverage		2	6.1
	Spread of frightening information by media	2	6.1
	Constant changes in information	1	3.0

Note. n* = statements in this category, ** % = percentage of GPs who stated an insight from this category.
